# Neurological Disorders Induced by Drug Use: Effects of Adolescent and Embryonic Drug Exposure on Behavioral Neurodevelopment

**DOI:** 10.3390/ijms25158341

**Published:** 2024-07-30

**Authors:** Olga Karatayev, Adam D. Collier, Stella R. Targoff, Sarah F. Leibowitz

**Affiliations:** Laboratory of Behavioral Neurobiology, The Rockefeller University, New York, NY 10065, USA; karatao@rockefeller.edu (O.K.); stella.targoff25@trinityschoolnyc.org (S.R.T.)

**Keywords:** maternal, alcohol, nicotine, cannabis, substance use disorders, neurological disorders, brain systems, neurodevelopment

## Abstract

Clinical studies demonstrate that the risk of developing neurological disorders is increased by overconsumption of the commonly used drugs, alcohol, nicotine and cannabis. These drug-induced neurological disorders, which include substance use disorder (SUD) and its co-occurring emotional conditions such as anxiety and depression, are observed not only in adults but also with drug use during adolescence and after prenatal exposure to these drugs, and they are accompanied by long-lasting disturbances in brain development. This report provides overviews of clinical and preclinical studies, which confirm these adverse effects in adolescents and the offspring prenatally exposed to the drugs and include a more in-depth description of specific neuronal systems, their neurocircuitry and molecular mechanisms, affected by drug exposure and of specific techniques used to determine if these effects in the brain are causally related to the behavioral disturbances. With analysis of further studies, this review then addresses four specific questions that are important for fully understanding the impact that drug use in young individuals can have on future pregnancies and their offspring. Evidence demonstrates that the adverse effects on their brain and behavior can occur: (1) at low doses with short periods of drug exposure during pregnancy; (2) after pre-conception drug use by both females and males; (3) in subsequent generations following the initial drug exposure; and (4) in a sex-dependent manner, with drug use producing a greater risk in females than males of developing SUDs with emotional conditions and female offspring after prenatal drug exposure responding more adversely than male offspring. With the recent rise in drug use by adolescents and pregnant women that has occurred in association with the legalization of cannabis and increased availability of vaping tools, these conclusions from the clinical and preclinical literature are particularly alarming and underscore the urgent need to educate young women and men about the possible harmful effects of early drug use and to seek novel therapeutic strategies that might help to limit drug use in young individuals.

## 1. Introduction

Neurological disorders denote conditions of the central and peripheral nervous systems that include neurodevelopmental, congenital and neurodegenerative disorders as well as substance use disorders (SUDs) and their co-occurring emotional conditions. The development of these neurological disorders results from complex interactions that occur between neurological systems and diverse contextual factors across the life course, including during embryonic development. This review focuses on the most commonly used drugs, alcohol, nicotine and cannabis, and the effects their use has on the development of SUDs, which involve excessive consumption of and motivation to seek drugs ranging from mild to moderate or severe [1] and are generally accompanied by emotional disorders such as an increase in anxiety, impulsivity, hyperactivity and depression [2,3]. As summarized below, clinical studies demonstrate that chronic overconsumption of these drugs in adults increases the risk of developing neurological disorders [4,5,6]. This negative outcome is found to similarly occur with the use of these drugs in younger and more vulnerable populations, including during adolescence and also in pregnant women, with their offspring at higher risk for developing such neurological disorders as SUDs and fetal alcohol spectrum disorder (FASD) [7,8,9,10,11,12,13]. With these adverse effects that drug use has on future generations, there is a growing global health concern, with more than 20% of women reporting drug use before pregnancy [14,15,16] and up to 14% reporting their use during pregnancy [17,18,19]. This increase in use of these drugs along with their misperceived safety has resulted, in part, from an increase in the availability of noncombustible nicotine products [20] and also of cannabis products that are now legalized in many jurisdictions and trend toward an increased content of tetrahydrocannabinol (THC), the primary psychoactive chemical extracted from cannabis plants, in the products being sold [21,22]. The rise in drug use creates a more urgent need to educate young individuals about the impact these frequently used drugs have not only on themselves but also on the brain and behavior of their future children.

**Figure 1:**
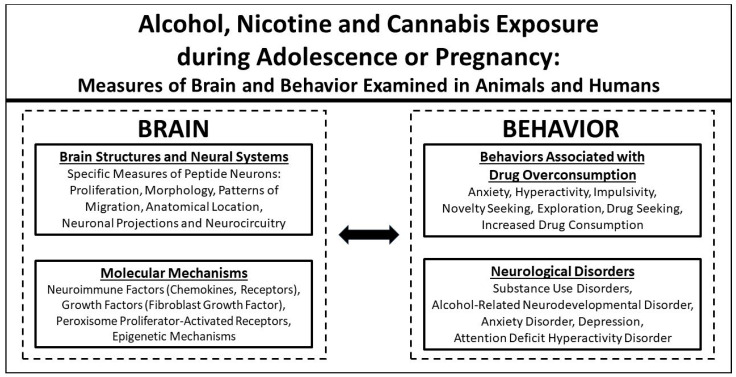
Summary of brain measures (neural peptides and molecular mechanisms) and specific behaviors and neurological disorders that are discussed in this review and affected by drug use during adolescence or pregnancy, contributing to overconsumption of the drugs.

**Figure 2:**
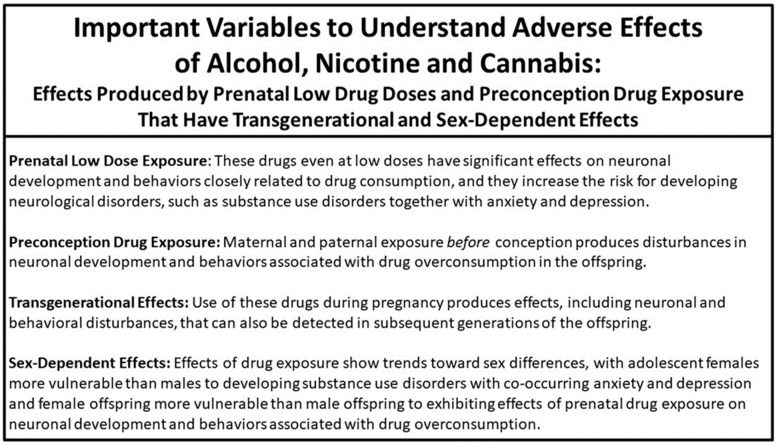
List of four specific variables closely examined in humans and animals in this review which are important for understanding the full impact of drug use on brain development and behavior.

Whereas the neurological disorders and related behaviors produced by the use of these drugs are well described in the clinical literature [4,5,6], the pragmatic and ethical barriers precluding research on human subjects has limited the information that can be obtained from clinical studies of drug effects on brain development. While research described below clearly shows drug-induced changes in the volume and connectivity of the human brain, animal studies provide more detailed information about specific neural systems, their projections and molecular mechanisms, that are dysregulated by drug use during adolescence and pregnancy and how this dysregulation relates to the specific behavioral, emotional and cognitive deficits involved in the neurological disorders. In addition to classical neurotransmitters such as dopamine, serotonin, norepinephrine, glutamate, GABA and endocannabinoids, these neural systems in animals and humans express various neuropeptides, including orexin, melanin-concentrating hormone, galanin and the opioids enkephalin and dynorphin, which are the focus of this review, and these neurochemicals expressed in different brain structures, such as the hypothalamus, preoptic area, amygdala, nucleus accumbens, caudate, hippocampus and cortical regions, are functionally involved in mediating motivation, reward, cognitive processes and emotional behaviors, including anxiety and depression, that increase the propensity to overconsume drugs [5,23,24,25,26]. This review describes studies investigating the effects produced by alcohol, nicotine and cannabis use at vulnerable ages, during adolescence and pregnancy, on the development of the different brain structures and neural systems expressing peptides and molecular mechanisms and on neurological disorders and related behaviors associated with drug consumption, as described in Figure 1. It first provides an overview of clinical studies in adolescents using these drugs and in the offspring of women reporting drug use during pregnancy, and this is followed by an overview of preclinical studies in which animals are exposed to these drugs during adolescence and pregnancy with greater experimental controls and more in-depth measures of the brain. This report then addresses several important variables listed in Figure 2, which are critical to our understanding the full impact that drug use at young ages has on neurological disorders such as SUDs and associated emotional conditions and also on specific neuronal systems in the brain known to mediate behaviors relates to these disorders. The results described here provide strong evidence indicating that the drug effects of alcohol, nicotine and cannabis on the brain and behavior can occur at low doses, with pre-conception drug use in females and males and in future generations. They further demonstrate that these three drugs are often quite similar in the effects that they produce, and females are more vulnerable than males to developing such disorders as SUDs with co-occurring emotional conditions and exhibiting prenatal drug-induced disturbances in the brain and behavior. These conclusions increase the urgency to communicate this information directly to young individuals and encourage efforts to reverse the growing divide which is forming between public attitudes suggesting that drug use is safe and the guidance of medical practitioners urging limited use in adolescents and during pregnancy [15,27].

## 2. Clinical Studies of Adolescent and Prenatal Drug Exposure on Neurological Disorders and Brain Development

With the goal of this research being to understand the harmful effects that early drug use during critical periods of human brain development (before age 25) has on brain mechanisms mediating the neurological disorders, longitudinal studies are particularly important for yielding impactful findings. Such clinical studies, however, face a host of challenges, confounding factors and practical/ethical issues that limit the information they can generate, and these include the complex and hard-to-control environmental, hormonal and genetic variables that affect brain development, the widely variable timing and amount of drug or polydrug use, the correlative nature and small sample size of these studies and the often inaccurate self-reporting and diagnosis of affected children [28]. Despite these limitations, recent clinical studies of brain mechanisms mediating drug-induced neurological conditions and related behaviors are now unequivocally demonstrating that the use of such recreational drugs as alcohol, nicotine and cannabis, particularly during vulnerable periods of adolescence and pregnancy, can strongly and negatively impact the brain and ultimately behavior.

### 2.1. Effects in Humans of Drug Use on Neurological Disorders

Studies in adults clearly demonstrate that the overconsumption of drugs dose-dependently increases the risk of developing various neurological disorders. Chronic alcohol consumption and even acute consumption involving binge drinking episodes cause deficits in cognition and memory and ultimately increase the possibility of developing Alzheimer’s and Parkinson’s [29,30], and chronic use of nicotine in adults similarly promotes the development of these neurodegenerative disorders in a dose-dependent manner [31,32,33]. Studies of drug use during adolescence, a critical period of significant structural, functional and neurochemical brain changes [34,35], also reveal adverse effects on behavior. The use of alcohol or nicotine in adolescents is associated with an increase in anxiety, impulsivity, inattention and cognitive deficits [36,37,38] and an increased likelihood of later drug abuse, with alcohol use before age 14 strongly linked to later development of SUDs [39,40] and teen smokers more likely to use other drugs in adulthood [41,42,43]. The use of cannabis during adolescence similarly has negative impact on behavior, adversely affecting cognitive function and increasing the risk of depression and autistic traits [44,45] and later use of other drugs [46]. Thus, chronic drug use during adolescence similar to effects in adults can promote the development of neurological conditions including SUDs.

Maternal consumption during pregnancy of alcohol, nicotine or cannabis also causes myriad adverse health outcomes in the offspring. Prenatal exposure to alcohol leads to neurodevelopmental disorders that continue well into adulthood and are associated with numerous behavioral problems, including greater anxiety, hyperactivity, depression, inattention, cognitive deficits and impairment of social functioning [8,9]. These disorders induced by alcohol use during pregnancy increase the offspring’s risk of developing SUDs, as suggested by evidence showing a greater reactivity of babies toward an alcohol odor [10], increased pleasantness of an alcohol odor in adolescents [11] and greater consumption of alcohol and other drugs such as cannabis in adolescents and adults [47,48]. Prenatal exposure to nicotine similarly leads to the development of neurological disorders, including attention deficit hyperactivity disorder and autistic traits [49,50], mood disorders [51,52,53,54,55] and emotional deficits including anxiety, depression and aggression [56], and it increases the propensity for later tobacco smoking and dependence, alcohol drinking and cannabis use [12,57,58]. Maternal consumption of cannabis during pregnancy also has behavioral effects in children and adolescents, including an increase in anxiety, depression, aggressive behavior and learning difficulties [59,60], greater impulsivity, hyperactivity and memory disturbances [59,60] and increased cannabis use during adolescence and young adulthood [13,61,62,63]. Together, these studies support the conclusion that drug use during pregnancy, whether it be alcohol, nicotine or cannabis, increases the propensity of the offspring to develop such neurological disorders as SUDs and their co-occurring emotional conditions.

### 2.2. Effects in Humans of Drug Use on Brain Development

Clinical studies applying different techniques demonstrate that these drug-induced neurological and neurodevelopmental disorders are accompanied by significant disturbances in brain development. Studies using non-invasive imaging techniques show that alcohol use during adolescence causes morphological abnormalities in the brain, including a decrease in the volume of specific limbic, hippocampal and cortical regions and the thickness of both white and gray matter in the cortex, along with disturbances in the connectivity within and between these brain regions [64,65,66]. In addition to these major changes induced by alcohol use during adolescence shown by magnetic resonance imaging, there is evidence for cellular, subcellular and molecular signaling mechanisms of neurodevelopment revealed by studies of the human-induced, pluripotent stem cell-derived brain organoid technique that recreates in detail the architecture and neurobiology of the brain. Investigations of a human organoid modeling an alcohol binge describe its multiple neurotoxic effects that involve neuron and astrocyte cell death, ultrastructural changes and mitochondrial dysfunction [67]. Neuroimaging studies of nicotine use at young ages also reveal harmful effects, with children exposed at 9–10 years old having lower whole brain measures in cortical areas and overall volume when examined 2 years later [68] and with nicotine use in adolescents causing a significant decrease in volume of the ventromedial prefrontal cortex and disturbances in neuronal connectivity within the corpus callosum [69]. Similarly, the use of cannabis disturbs brain structures of adolescents, producing a reduction in the volume of gray matter [70,71,72], white matter [73] and hippocampus [74] and a decrease in the functional connectivity between different cortical areas [44]. This evidence for changes induced by drug use at young ages clearly demonstrates the adverse effects that recreational drugs such as alcohol, nicotine and cannabis can have on specific brain areas likely to have marked impact on behavior.

Prenatal exposure to these drugs produces changes in the trajectory of development of the human fetal brain as well as the children and adolescent offspring. Analyses via different imaging techniques reveal multiple, alcohol-induced macrostructural and microstructural abnormalities, including abnormal thickness of the cortex and white matter integrity in the corpus callosum [75,76], disturbances in functional connectivity between different brain regions [77,78] and heterotopias in the brains of children with FASD [7]. They also identify neural correlates of specific behavioral disturbances induced by prenatal alcohol exposure, with changes in functional connectivity in children reliably predicting negative cognitive outcomes [77] and a reduced volume of brain regions in adolescents associated with a decrease in performance on verbal memory tests [79]. Studies using human brain organoids to model prenatal alcohol exposure have described a wide range of adverse effects, with alcohol causing unhealthy organoids at the cellular, subcellular and gene expression levels and producing mitochondrial dysfunction [67], attenuating neurite outgrowth and skewing neural maturation [80], and disturbing cell cycle and proliferation, while additionally altering post-translational histone modifications and chromatin accessibility that impair cAMP and calcium signaling, glutamatergic synaptic development, and astrocytic function [81]. Prenatal nicotine exposure similarly produces significant changes in brain development, with brain imaging studies of preadolescent or adolescent offspring revealing smaller total brain volume; reduced gray and white matter volume; decreased size of specific brain structures, including the amygdala and frontal lobe [82,83,84]; and a formation of Purkinje cell heterotopias that may contribute to SUDs in the offspring [85]. As revealed by the human brain organoid-on-a-chip system, exposure to nicotine causes premature neuronal differentiation, disrupts cortical development, impairs neurogenesis and alters the migration of neurons [86]. Prenatal exposure to cannabis similarly disturbs brain development in the fetal offspring, altering brain morphology [87] and gene expression of the reward-related opioid and endocannabinoid systems [88,89,90] while having long-lasting effects on neuronal function by altering synaptic plasticity [91]. A study using human brain organoids to model prenatal cannabis exposure describes a reduction in neuronal maturation as well as impaired neurite outgrowth [92]. This clinical evidence demonstrates the profound effects that alcohol, nicotine and cannabis use during adolescence and pregnancy can have on developing brain systems and their connectivity, changes that are likely to contribute to the disturbances in neurodevelopmental behaviors including later overconsumption of these drugs.

## 3. Animal Studies of Adolescent and Prenatal Drug Exposure on Different Behaviors and Brain Development

While clinical studies clearly show that drug use during adolescence and pregnancy increases the risk of developing neurological disorders and produces defects in brain volume, structure and gene expression, studies in animals with stronger experimental controls and a wider range of measures in the brain allow one to identify and characterize in greater depth and over tine the disturbances that occur in specific, closely studied neuronal systems. The preclinical results presented here, in addition to revealing effects of early drug exposure on different behaviors related to the neurological disorders described in clinical studies, provide more detailed information about the changes that occur in these neuronal systems and how these disturbances are closely and possibly causally related to the neurological conditions.

### 3.1. Effects in Animals of Drug Exposure on Different Behaviors

As in human studies, exposure to drugs in animals during critical periods of brain development, such as during adolescence and the embryonic period in rodents (before 3 months) and zebrafish (around 45 days), has adverse effects on a range of behaviors related to neurological conditions and the use of drugs. For example, alcohol consumption in rodents during adolescence, similar to effects in adults [93,94], causes a variety of behavioral disturbances, including an increase in anxiety, impulsivity, hyperactivity, and cognitive impairments along with greater consumption of alcohol [93,94,95]. Prenatal exposure to alcohol during pregnancy has more severe effects on the offspring, which involve a wide range of behavioral changes that start early in life and last long into adulthood [96,97] and parallel the effects described above in clinical studies. Similar to FASD in humans, these behavioral changes in rodent offspring include an increase in locomotor activity, anxiety, exploration and alcohol seeking behavior, along with an increase in voluntary alcohol consumption during adolescence that persists into adulthood [97,98,99,100] and vulnerability to developing addiction to other drugs such as cocaine [101]. Notably, these diverse behavioral disturbances induced by prenatal alcohol exposure in rodents are confirmed in the zebrafish model and similarly observed in larval and juvenile fish as well as adults after exposure of the embryo to alcohol in the water [102,103].

Exposure to nicotine during adolescence also induces a range of behavioral disturbances in rodents, including an increase in anxiety- and depressive-like disorders [104] and impulsivity in adults [105], and these effects serve as a gateway to the use of other drugs [106], with nicotine-exposed rodents found to be more sensitive to the locomotor-activating effects of cocaine [107] and more susceptible to the co-use of nicotine and alcohol during adolescence [108] and the self-administration of alcohol and cocaine in adults [109,110]. As with alcohol, prenatal exposure to nicotine leads to behavioral changes related to the attention deficit hyperactivity disorder, including an increase in locomotor activity as well as anxiety-like and impulsive behaviors in adolescent rats [56,111,112,113], and these behaviors are closely associated with an increase in preference for nicotine odor at postnatal and adolescent ages [114] and a greater consumption and self-administration of nicotine and alcohol in adolescent offspring [113,115]. Rodent studies of cannabis exposure during adolescence similarly reveal behavioral disturbances in multiple domains, including emotionality, cognition and memory [116,117], and these behavioral effects again have symptoms related to neurological disorders [118], serve as a gateway to the use or self-administration of other drugs of abuse [119,120,121] and are long-lasting into adulthood [122]. Prenatal exposure to cannabis in rodents produces behavioral changes in the offspring, including deficits in cognition, emotionality and social interaction evident during adolescence as well as adulthood [123,124,125]. It also causes an increase in anxiety, hyperactivity and impulsivity during adolescence [125,126] and induces drug seeking behavior as indicated by an increase in morphine and heroin self-administration in adults [127,128]. Together, these animal studies, consistent with human reports, provide strong evidence that adolescent and prenatal exposure to alcohol, nicotine and cannabis has major impact on a wide range of behaviors related to various neurological conditions including SUDs.

### 3.2. Effects in Animals of Drug Exposure on Brain Development

Along with these behavioral effects of early drug exposure, preclinical studies provide evidence for marked effects on the brain and particular neuronal systems that control drug-related behaviors. Exposure to alcohol in adolescent rodents, in addition to disturbing the integrity of the white matter and volume of gray matter, causes a decrease in neurogenesis and myelination integrity in specific areas and disturbs the functional connectivity between these regions [129,130,131]. Prenatal exposure to alcohol has even more severe and diverse effects in the offspring, including disturbances in brain structures, brain volume, programmed cell death, neurogenesis, neuronal differentiation and migration, synaptic connections, and other aspects of brain development and neuronal function [132,133,134]. It also disturbs specific neurotransmitter systems involved in drug-related behaviors, generally reducing the levels and expression of dopamine, norepinephrine, serotonin, glutamate and GABA throughout the brain [135,136], while at low-moderate doses it can increase the number of neurons expressing such peptides as orexin, melanin-concentrating hormone, galanin and the opioid enkephalin in the hypothalamus in close association with an increase in voluntary consumption of alcohol [95,137]. Once again, this stimulatory effect on peptide neurons in rodents is similarly observed in larval and juvenile zebrafish exposed as embryos to alcohol in the water, and this effect is accompanied by similar behavioral changes including an increase in voluntary alcohol consumption [138].

As with alcohol, adolescent exposure to nicotine in rodents causes disturbances in neural systems, including perturbations in the cholinergic system, dendritic morphology, and functional connectivity between brain regions [105,139,140]. Further, maternal consumption of nicotine during pregnancy, in addition to reorganizing the neuroanatomical morphology and altering the neuronal connectivity and function in the fetal brain [141,142], stimulates in rodent offspring the expression of hypothalamic peptide neurons that persists throughout puberty and increases the proliferation of their neural progenitor cells [115,143,144]. The use of cannabis during adolescence similarly leads to structural changes in the rodent brain, such as a loss of gray matter in the hippocampus and cortex, neural changes in the amygdala, and alterations in functional connectivity between different brain regions, effects that likely alter the functioning of various neurochemical systems [123,145,146]. Prenatal cannabis exposure in rodents produces more severe negative neurodevelopmental outcomes in the offspring, including a decrease in brain volume in fetuses that persists into adulthood [147] and long-lasting functional neuronal changes such as disturbances in neurotransmitter release and modulation of brain plasticity in neural pathways underlying motivation, cognition and behavior regulation [148,149,150]. Studies of the opioid peptide systems in rodents demonstrate that adolescent exposure to THC stimulates the expression of dynorphin neurons in the nucleus accumbens [151], and prenatal exposure to THC after an initial reduction in enkephalin mRNA in the accumbens causes an increase in enkephalin expression in the amygdala of adult offspring [128]. Thus, in addition to confirming in rodents as well as zebrafish the effects of early drug exposure on brain development as described in humans, these animal studies clearly demonstrate that alcohol, nicotine and cannabis are similar in having adverse effects on specific brain areas that are likely to contribute to the drug-induced disturbances in behaviors related to drug overconsumption.

## 4. Effects at Low Doses of Prenatal Drug Exposure on Neurological Behaviors and Brain Development

With preclinical studies revealing similar effects to clinical studies of adolescent and prenatal drug exposure on behavior and the brain, we can now focus on the four important questions that need to be addressed to fully understand the overall impact that drug use can have on future progeny. The first, frequently asked question is whether there is any dose of the recreational drugs, alcohol, nicotine or cannabis that can be considered safe and is unlikely to produce adverse effects on brain development and subsequent behavior. While this question has been extensively debated by many scientists and clinicians [133,152,153], the answer is still unclear, leading investigators to conclude that there is really no dose of these drugs that is certain to be safe, with lower doses likely to have smaller and fewer effects than moderate-to-high doses [154,155,156]. The studies described here which test a variety of doses clearly demonstrate that these drugs at low doses, in fact, can have significant impact on embryonic brain development and can induce specific behavioral changes in the offspring that are related to particular neurological disorders.

### 4.1. Effects in Humans of Prenatal Drug Exposure at Low Doses on Behavior and Brain Development

Clinical studies of prenatal exposure to low doses of alcohol, nicotine or cannabis reveal a wide range of significant changes in the offspring. While a threshold for safe drinking of alcohol during pregnancy has yet to be established [133], there is evidence that low amounts of alcohol use during pregnancy alter a large number of developmental parameters in the embryo, which in turn may lead to significant behavioral changes early in life that continue through adolescence and into adulthood. Prenatal exposure to the lowest levels of alcohol produces what is called alcohol-related neurodevelopmental disorder (ARND), a less severe but still significant form of FASD characterized by a history of prenatal alcohol exposure without the cardinal facial dysmorphology [157]. There is clinical evidence that these low alcohol levels cause an increase in anxiety and depression in infants [158] and behavioral and cognitive impairments in children [133,159,160], supporting the phrase that “no alcohol dose is safe during pregnancy” [154,156]. Taking just one drink each day early in pregnancy is shown to increase alcohol consumption in the adult offspring [161], while 3 or more drinks per occasion increases their risk of developing an alcohol use disorder later in life [162]. Prenatal exposure to low doses of nicotine also produces significant behavioral changes in the offspring, causing in children and adolescents an increase in locomotor activity and internalizing behaviors such as anxiety and depression in conjunction with disturbances in cognitive abilities and scholastic achievements [163,164,165] as well as an increased risk for tobacco smoking later in life [57,58]. Even secondhand smoke exposure during pregnancy produces significant impairments in fine motor skills and poorer communication in the offspring [166]. Prenatal exposure to cannabis at low doses is also unsafe for the offspring, with clinical studies showing it to produce a myriad of behavioral and cognitive disturbances that include a decrease in attention span, verbal/memory processing and cognitive performance [167] and an increased risk of greater cannabis use in adulthood [62]. These clinical studies support the idea that no amount of drug use during pregnancy is safe, with small amounts found to have some adverse effects on the behavior of the offspring.

There is clinical evidence showing that drug use at low doses during pregnancy can also impact brain development in the offspring, producing alterations in the structure and function of specific areas that may contribute to the drug-induced neurological disorders. In children, prenatal exposure to the lowest levels of alcohol significantly reduces the volume of gray matter in the caudate nucleus and the cingulate, temporal and frontal gyri, and it alters the functional connectivity within these brain areas [133,168,169,170]. While clinical studies of low nicotine or cannabis doses during pregnancy are lacking, low smoking exposure during adolescence is found to cause structural changes in the brain, including alterations in the volume of the ventromedial prefrontal cortex and the neuronal connectivity of the corpus callosum [69]. In addition, studies using three-dimensional human brain organoids demonstrate that low alcohol doses comparable to an alcohol binge cause neuroapoptosis, mitochondrial dysfunction and ultrastructural cell changes such as degenerated synapses [67]. Nicotine exposure at low doses also produces cell apoptosis and disrupts the differentiation of cortical neurons [86]. Together, these clinical studies present evidence demonstrating that maternal use of these drugs at low doses during pregnancy has adverse effects on the brain as well as subsequent behavior of the offspring.

### 4.2. Effects in Rodents of Prenatal Drug Exposure at Low Doses on Behavior and Brain Development

Studies in rodents, using a wide range of doses and schedules of drug administration, provide clear evidence, consistent with clinical studies, for significant effects on the offspring’s behavior of prenatal exposure to low doses of alcohol, nicotine or cannabis. For example, prenatal alcohol exposure at relatively low doses for only 5 days has ARND-like effects in the pre-weanling and adolescent offspring, including an increase in locomotor activity, anxiety, exploration, alcohol seeking behavior and alcohol consumption [95,102,171], and these effects are shown to persist into adulthood [172,173], sometimes without disturbances in cognition, spatial learning and memory performance [173]. Low doses of nicotine exposure during pregnancy in rodents also cause significant disturbances in different behaviors during adolescence and adulthood, such as greater anxiety-like behavior and locomotor activity levels [174,175], selective deficits in learning an avoidance response [176] and increased self-administration of nicotine and alcohol [115] and other drugs such as cocaine and fentanyl [177]. Prenatal exposure to cannabis at low doses also has strong behavioral effects in the offspring, causing atypical locomotor activity; emotional disturbances, such as increased anxiety; impairments in cognition and attention; and an increase in susceptibility to drug use [167,178,179]. These studies in rodents, consistent with clinical studies, provide compelling evidence that low drug doses during pregnancy can affect a large range of behaviors in the offspring which include an overconsumption of drugs that persists into adulthood.

Prenatal drug exposure in rodents also produces changes in the structure and function of the offspring brain at low doses. While moderate-to-high doses of alcohol during pregnancy have severely profound adverse effects throughout the brain on essentially all measures including cell survival, apoptotic cell death and the birth, proliferation, maturation and projections of neurons [180,181,182], low doses of alcohol produce effects that are smaller and anatomically localized and may even involve a stimulation of specific neuronal systems and their neurocircuitry. While rodent studies with short periods of low alcohol doses show no significant changes in cell survival and apoptosis, they reveal a significant increase in cell proliferation and neurogenesis in particular brain areas, including the hypothalamus, nucleus accumbens and amygdala and also in the proliferation of specific hypothalamic peptide neurons known to promote the overconsumption of drugs [115,173,183,184]. These effects on peptide neurons in the offspring brain may be highly anatomically specific, with alcohol shown to increase the density of enkephalin-expressing neurons specifically in the core but not the shell of the nucleus accumbens, again while producing no changes in cell death [137]. In addition to affecting the number of peptide-expressing neurons, low doses of prenatal alcohol exposure alter the migration of these neurons [185], causing neurons with orexin and melanin-concentrating hormone to be ectopically expressed outside their normal hypothalamic location and further anterior in the nucleus accumbens core and ventromedial caudate putamen [102]. These low doses also alter the morphological characteristics of these peptide neurons, sometimes leading them to be smaller in size and to have fewer processes [102]. Studies of other neuronal systems in the offspring show low doses of alcohol to cause aberrant migration and ectopic expression of GABA neurons [186,187] and induce cortical neurons to form heterotopias [188], consistent with the heterotopias found in the brains of children with FASD [7]. Further, prenatal alcohol exposure at low doses affects the neurocircuitry and thus function of neurons in specific brain areas, increasing the number of processes emanating from the soma of orexin peptide neurons in the hypothalamus [102], stimulating the length and complexity as well as number of projections from neurons in the hippocampus [189] and altering the dendritic morphology while increasing synaptic spine density along the apical dendrites of pyramidal neurons in the amygdala [173].

Studies in rodents involving prenatal exposure to low doses of nicotine and cannabis similarly provide evidence for changes in specific aspects of neuronal development in particular brain areas of the offspring. Whereas moderate-to-high doses of nicotine suppress neurodevelopment throughout the brain, as shown by a decrease in cell number and survival and an increase in apoptotic cell death along with a reduction or no change in peptide neurons [190,191,192], low doses of nicotine are found to have specific stimulatory effects on the development of neurons [115]. These include an increase in neurogenesis with no change in apoptotic cell death and an increase in the number of newly generated, peptide neurons expressing enkephalin or orexin in the hypothalamus and amygdala of pre-weanling and adolescent offspring, effects that are positively related to a later increase in consumption of nicotine as well as alcohol [115,193]. These changes in development of peptide neurons are again accompanied by morphological changes, including alterations in dendritic branching, dendritic length, and spine density in the nucleus accumbens and medial prefrontal cortex [141,194]. Prenatal exposure to cannabis at low doses similarly affects brain development in the offspring. While the effects of cannabis on neurogenesis, neuronal cell death and peptide neurons have yet to be characterized, there is evidence that low doses of prenatal THC exposure stimulate the expression of enkephalin in the amygdala of adult offspring [128], while low cannabis doses reduce the volume of the diencephalon [147] and dopamine receptor expression in the amygdala and ventral striatum [89,90,195]. Exposure to low cannabis doses during adolescence similarly stimulates the expression of the opioid peptides, dynorphin [151] and enkephalin in the nucleus accumbens of adult rats [119,196], underscoring the significant effects that low doses of this drug have on opioid systems. Further, prenatal cannabis exposure affects the developmental route of neuronal brain regions while having functional consequences [197,198], with low–moderate doses shown to alter the migration of neurons in the brains of the embryo [199] and juvenile offspring [147,148]. Low doses of prenatal cannabis also cause extensive molecular and synaptic changes in midbrain dopaminergic neurons that contribute to increased behavioral sensitivity to acute THC exposure during early adolescence [126]. Together, these studies in rodents demonstrate how low doses of these drugs significantly alter the development of the offspring brain, consistent with results described in clinical reports.

### 4.3. Effects in Zebrafish of Embryonic Drug Exposure at Low Doses on Behavior and Brain Development

Although not as well established as rodent models, zebrafish have been successfully used for modeling neurobehavioral phenotypes related to drug overconsumption and investigating the behavioral effects produced by exposure of the embryo to drugs placed in the water [200,201,202,203]. As in rodent studies, embryonic drug exposure in zebrafish alters behaviors in a dose-related manner, with low doses often having stimulatory effects while higher doses have suppressive effects as reported for locomotor activity after embryonic exposure to alcohol [204], nicotine [205], and cannabinoids [206]. As summarized in several comprehensive reviews [23,204,207,208,209,210,211], the behavioral changes produced in zebrafish by embryonic alcohol exposure at low concentrations having no effects on gross morphology are ARND-like behaviors, similar to those produced in rodents by prenatal alcohol exposure at low doses as described above. These behavioral effects in zebrafish involve disturbances in emotion, motivation, perception and associative learning processing, and they are accompanied by an increase in the reinforcing effects of alcohol, alcohol seeking behavior and preference for alcohol [212]. Embryonic exposure to nicotine in zebrafish also alters their subsequent behavior, stimulating active nicotine seeking at a low concentration while producing nicotine avoidance at a high concentration [213]. With zebrafish having a major advantage of allowing behaviors to be tested very early in development, additional studies involving behavioral measurements at just 5 days after fertilization demonstrate that embryonic exposure to alcohol at a low dose causes a range of behavioral changes, which include an increase in anxiety, exploration, locomotor activity, impulsivity and novelty seeking followed by an increase in voluntary alcohol consumption [103,214] and are similar to those described in pre-weanling rats prenatally exposed to a low alcohol dose [95,102]. Embryonic exposure to nicotine is also shown to stimulate anxiety-like behavior in larval zebrafish while reducing habituation to an acoustic startle stimulus [215,216], and embryonic cannabinoid exposure has similar effects on this startle response [217]. These early behavioral disturbances induced in zebrafish by embryonic drug exposure are long lasting and persist into older ages, with adult zebrafish showing an increase in risk-taking behavior after embryonic exposure to alcohol [208,218] or cannabinoids [219] and also in startle responses after embryonic exposure to nicotine [220].

Our understanding of the effects of embryonic drug exposure on brain development has been greatly advanced by studies in zebrafish, which have major advantages in being transparent as embryos and larvae and highly genetically tractable, allowing methods of transgenesis to be easily established for labeling neuronal populations with fluorescent proteins [221,222,223]. Exposure to a low alcohol dose increases the embryo’s alcohol concentration to levels equivalent to those observed in humans at birth prenatally exposed to alcohol [224,225,226], and zebrafish at a young age are found to have a well-conserved neurocircuitry with neuronal targets of nicotine and cannabis such as nicotinic acetylcholine [227] and endocannabinoid receptors [228] and specific biological mechanisms similar to humans that are required to metabolize nicotine into cotinine [229]. Further, the effects in zebrafish of embryonic drug exposure on the development of neurons and their neurocircuitry as summarized in several reviews [202,204,209,210,211,230] are very similar to those described in rodents. For example, embryonic exposure to alcohol at relatively low doses that have no morphological effects increases the proliferation and differentiation of neurons in the developing hypothalamus [103,231], while moderate–high doses that produce morphological changes decrease neuronal differentiation [232] and the number of neurons and neural precursor cells [233]. While the neurotransmitter systems and particularly the monoamines are the most well studied in zebrafish and are often found to be reduced by embryonic drug exposure [234,235,236], recent studies of the large peptide-expressing neurons, which are well conserved in zebrafish and have a natural functional relationship with drug-related behaviors including locomotor activity, anxiety, sleep/wake cycle, consummatory behavior and drug intake [23], have greatly expanded our knowledge of the diverse and subtle effects that embryonic drug exposure has at low concentrations on brain development. Embryonic alcohol exposure produces long-lasting changes in these peptide neurons, as revealed by measures of their number, morphology, proliferation, migration, location and colocalization of different peptides [102,103,138,237].

**Figure 3:**
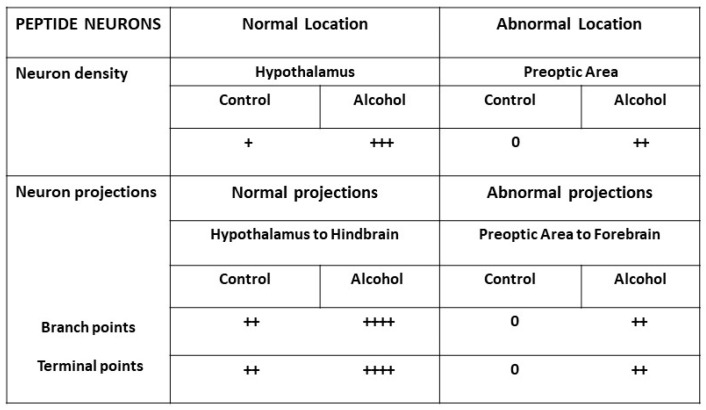
Effects of embryonic alcohol exposure at low doses on development of orexin peptide neurons, which involve disturbances in their location and projection areas in the hindbrain and forebrain of zebrafish, as described in the text. Neuron density: Compared to control which has low (+) peptide neurons in the hypothalamus or no (0) peptide neurons in the preoptic area, alcohol increases the number of neurons in the hypothalamus (+++) and the preoptic area (++). Neuron projections: Compared to control which has moderate (++) hypothalamic projections to the hindbrain or no (0) preoptic projections to the forebrain, alcohol increases the branch points and terminal points in normal hypothalamic projections to high levels (++++) in the hindbrain and in abnormal preoptic projections to moderate levels (++) in the forebrain.

For example, as recently described [102,238] and illustrated in Figure 3, alcohol at a low dose increases the density of orexin peptide neurons in their normal hypothalamic location, as well as their size and number of processes, and it causes other peptide neurons to migrate outside their normal location and become abnormally located further anterior in the preoptic area. In addition, alcohol at low doses stimulates the projections of normally located peptide neurons that innervate the hindbrain, increasing their branch and terminal points in the lower brainstem areas known to mediate locomotor activity and sleep/wake behaviors, and it causes projections of the abnormally located preoptic neurons to develop branch and terminal points, innervating more anterior forebrain regions known to promote behaviors related to drug overconsumption. There is further evidence in zebrafish that embryonic exposure to nicotine has long-lasting effects on the axonal pathfinding of secondary motor neurons, changes that persist into adulthood and are reversed by treatment with nicotinic receptor antagonists [239,240]. Moreover, embryonic cannabinol exposure is found to reduce the number of axonal branches of motor neurons, while THC exposure decreases the axon diameter of reticulospinal neurons in the hindbrain [241,242,243].

Zebrafish are also ideally suited to investigate whether the changes in specific neuronal populations caused by low drug doses are directly, and possibly causally, related to the behavioral disturbances involved in the overconsumption of drugs. The advanced techniques being applied in zebrafish research, including optogenetic and chemogenetic neuronal activation and silencing, calcium imaging, targeted neuronal laser ablation, and genetic manipulations with tools such as CRISPR and morpholino oligonucleotides, provide evidence to support this possibility. For example, consistent with rodent studies showing optogenetic and chemogenetic activation specifically of orexin neurons to stimulate locomotion, anxiety and impulsivity [244,245,246], a recent report in zebrafish using optogenetic activation of these peptide neurons [214] shows this direct stimulation to produce behavioral changes similar to those induced by embryonic alcohol exposure at a low dose. This provides support for the role of these neurons in mediating the behaviors, which include an increase in locomotor activity, anxiety, exploration, motor impulsivity, novelty seeking and alcohol seeking observed in larval fish and a later increase in voluntary alcohol consumption observed in juvenile fish. There is another report using direct laser ablation of these peptide neurons [102], which demonstrates that the drug-induced increase in locomotor behavior is completely blocked by ablating the specific orexin neurons that are ectopically expressed outside the hypothalamus, supporting the importance of this abnormal neuronal subpopulation in mediating the behavioral disturbances. Further, use of the recently developed calcium indicator CaMPARI to measure neuronal activation in free-swimming larvae as it relates to the drug-induced behavioral changes demonstrates that embryonic cannabidiol and THC exposure causes a reduction both in whole-brain neuronal activity and in locomotor activity, effects partially blocked by antagonists of the cannabinoid receptor [247]. Also, morpholino knockdown of a nicotinic acetylcholine receptor is shown to block the nicotine-induced increase in locomotor activity while having no effect of its own on spontaneous motor activity [239]. Together, these studies illustrate how the smaller brain of the zebrafish allows one to quantify and characterize in greater depth a specific and entire neuronal population. Using advanced techniques, they provide new information which reveals the diverse and sometimes subtle effects that embryonic drug exposure at low doses has on different measures of specific neurons and their neurocircuitry and also demonstrate how these effects mediate the particular drug-induced disturbances in behaviors that contribute to later overconsumption of these drugs.

### 4.4. Molecular Mechanisms Mediating Effects of Embryonic Low Dose Drug Exposure on Behavior and Brain of the Offspring

With animal studies of embryonic drug exposure at low doses identifying changes in specific neurons that may contribute to the behavioral disturbances in the offspring involved in neurological disorders such as SUDs, we describe here particular molecular mechanisms which have been investigated in the hypothalamic peptide neurons and may underlie drug-induced neuronal changes as well as their behavioral consequences. These mechanisms include growth factors, neuroimmune systems and peroxisome proliferator-activated receptor, and they also involve epigenetic mechanisms which are described below. In addition to studies showing brain-derived neurotrophic factor to have an important function in neuronal proliferation and differentiation [248] and to be linked to neuronal changes and cognitive deficits induced in the offspring by prenatal/neonatal drug exposure [147,249,250,251,252], there are recent reports examining fibroblast growth factor-2 and its function in mediating the effects of drug use on peptide neurons. This growth factor, which is known to stimulate neuronal development [253,254] and have circulating levels in children that are increased by prenatal alcohol exposure [255], is shown to produce an increase in alcohol and nicotine consumption in rodents and to have a role in the development of other neurological conditions including anxiety and depression in children [253,256,257,258]. Studies in rodents demonstrate that fibroblast growth factor-2 and its receptor are co-expressed in the hypothalamic peptide neurons and are markedly stimulated in the offspring by prenatal alcohol exposure at a low dose [259]. Moreover, peripheral injection of this growth factor in pregnant rats mimics these stimulatory effects of prenatal alcohol exposure, not only on the peptide neurons but also on the behaviors induced by alcohol [259]. The expression of this growth factor in adolescent rats is similarly increased in the hippocampus by neonatal exposure to nicotine [252] and in the nucleus accumbens and prefrontal cortex by administration of cocaine [260]. Together, these studies provide evidence directly supporting the involvement of growth factors in the effects of prenatal drug exposure on specific neuronal populations and drug-associated behaviors.

Neuroimmune signaling, involving such proinflammatory chemokines as CCL2 and CXCL12 and their respective receptors CCR2 and CXCR4, is believed to be a key mediator in the molecular pathways positively linking the immune system to neuronal development and function [261,262,263] and also to have a role in the development of various neurological conditions including depression, addiction, attention deficit hyperactivity disorder and schizophrenia [264,265,266,267]. These chemokine systems are detected in the peptide neurons that express orexin and melanin-concentrating hormone [95,268,269], and they are involved in stimulating the proliferation and migration of neurons and the development of their projections [270,271]. Drug exposure stimulates these proinflammatory chemokine systems in the offspring brain, which in turn are shown to affect neuronal development and behavior of the rat as well as zebrafish offspring [171,272,273,274]. Embryonic alcohol exposure at a low dose increases these chemokines, not only in the hypothalamic peptide neurons but also in their neuroepithelial progenitor cells that further promote neurogenesis, and these effects are mimicked by CCL2 after peripheral administration in pregnant rats or central administration directly into the embryo brain and are blocked by peripheral administration of chemokine receptor antagonists in pregnant rats [95,275,276]. Further, as in rats, the overexpression of CXCL12 in the zebrafish brain, similar to alcohol exposure, has a stimulatory effect on the expression of peptide neurons [272], supporting a cross-species involvement of these neuroimmune factors in mediating the neuronal as well as behavioral effects of alcohol exposure. There is further evidence that prenatal nicotine exposure stimulates proinflammatory chemokines such as CCL2 in rodents [277], prenatal exposure to environmental tobacco smoke increases plasma CCL2 in infant primates [278], and environmental tobacco smoke elevates such proinflammatory markers as CCL2 in the brain of adult rats [279]. Prenatal cannabis exposure similarly stimulates proinflammatory markers in the fetal brain as shown with injection of THC [280]. These studies demonstrate how the neuroimmune system may have a key role in mediating drug-induced changes in neuronal development.

Peroxisome proliferator-activated receptors (PPARs), a superfamily of ligand-binding nuclear receptors with three isoforms that are master regulators of adipogenesis, perform a broad range of functions related to cellular development and the differentiation, maturation and developmental plasticity of neurons, and thus have a crucial role in the transition from embryo to fetus and in the process of remodeling and improving the match between phenotype and the environment [281]. These receptors, also investigated in the management of neurodegenerative disorders and addiction [282], are major targets of cannabinoids [283] and are shown to be modulated by prenatal exposure to drugs [284,285]. Studies of hypothalamic neuronal development in rodents demonstrate that PPARs are strongly expressed in the different peptide neurons involved in controlling drug-related behaviors, and the expression and development of these neurons are found to be regulated by PPARs [27,286,287]. This is demonstrated in vitro, with enkephalin transcription in hypothalamic neurons significantly stimulated by a PPAR antagonist with the opposite produced by an agonist [286], and also in vivo, with the proliferation of hypothalamic peptide neurons and their co-expression of PPAR stimulated in the offspring by prenatal exposure to consumption of food rich in fat [287]. Altogether, these studies provide examples of specific molecular mechanisms, involving growth factors, chemokines and PPARs, which are known to control neuronal development and shown here to be affected by prenatal manipulations and, in turn, to influence the development of specific peptide neurons that promote drug overconsumption. Further investigations of the molecular signaling in particular neuronal populations such as peptide-expressing neurons may help to establish more targeted therapeutic strategies for attenuating the adverse effects that drug use during pregnancy can have on embryonic development and subsequent behavior.

## 5. Pre-Conception Drug Effects on Behavior and Brain Development of Offspring in Humans and Animals

With the pre-conception period providing a unique opportunity for optimizing with proper care the health of both women and men and consequently their children, we are led to address the second important question, as to whether the use of alcohol, nicotine or cannabis before pregnancy effects the offspring which had no direct exposure to the drug. Here we describe both clinical and preclinical studies investigating, in both females and males, the impact of pre-conception drug use. The findings provide strong evidence indicating that paternal as well as maternal drug use prior to conception has strong, deleterious effects on the behavior and brain development of the offspring.

### 5.1. Pre-Conception Drug Use in Females Affecting the Behavior and Brain Development of Their Offspring

In addition to the effects produced by maternal drug use during pregnancy of alcohol, nicotine or cannabis at low doses, there is clinical evidence that maternal health involving drug use before conception also markedly affects the pregnancy and health outcome of the offspring. Women consuming alcohol during the pre-conception period are found to have lower conception rates, increased risk of miscarriage and neural tube defects and reduced embryonic growth trajectories of the offspring [288,289], and this pre-conception alcohol consumption at moderate-to-high levels is additionally shown to cause cognitive deficits in their children, including lower IQ scores, verbal test scores and overall attention scores [290,291]. These results are consistent with studies in rodents, demonstrating that maternal pre-conception alcohol intake retards neuronal development, alters embryo morphology and impairs blastocyst hatching [292], and it also stimulates certain behaviors in the offspring such as locomotor activity, impulsivity and exploration while impairing spatial memory and attention [293,294]. In addition, pre-conception alcohol is shown to increase the consumption of calorie-rich food while altering the dopaminergic system [295], and it affects the hypothalamic–pituitary–adrenal axis function as well as the signaling elements and expression of genes associated with anxiety, depression and disturbances in social behaviors [296,297].

Clinical studies demonstrate that maternal use of tobacco before pregnancy, like alcohol, has adverse effects on the pregnancy and health of the offspring, negatively impacting placental weight and increasing the risk of pregnancy complications [298]. Furthermore, maternal pre-conception smoking as well as second-hand smoke exposure increases the risk of developing traits of autism spectrum disorder in the offspring [299,300]. Similar results are described in rodent studies, showing pre-conception nicotine exposure in females to cause disturbances in the offspring’s cognitive ability and motor development [301,302,303]. Further, maternal use of cannabis before pregnancy impairs placental development, leading to high incidents of preterm births and low birth weight [304,305], and it increases externalizing but not internalizing problems in the offspring [305]. Rodent models of female pre-conception cannabis exposure demonstrate both an increased effort in the offspring to self-administer heroin along with abnormal behaviors during heroin withdrawal [306] and an increased preference for morphine and responses to morphine challenges along with greater expression of an opioid receptor in the nucleus accumbens [307,308]. Together, these studies demonstrate that maternal use of alcohol, nicotine or cannabis before pregnancy negatively impacts the pregnancy outcome as well as the mental, emotional and physiological health of the offspring.

### 5.2. Pre-Conception Drug Use in Males Affecting the Behavior and Brain Development of Their Offspring

Whereas the primary focus of many studies has been to investigate the consequences of maternal drug use, there is compelling evidence that paternal pre-conception use of recreational drugs, which has markedly increased in men of reproductive age, also has adverse consequences in their offspring [309,310]. Clinical studies demonstrate that paternal pre-conception alcohol use increases the risk of intrauterine growth restriction and insulin hypersensitivity and disturbs the growth and long-term metabolic programming of the offspring [311], and it also produces an increased risk of developing such neurological conditions as attention deficit hyperactivity disorder [312]. Moreover, paternal alcohol use dose-dependently increases the potential of child behavioral problems such as anxiety, depression and sleep disturbances [313,314], with evidence showing the sons of male alcoholics to have a markedly increased risk for developing alcohol abuse and neurotic personality profiles [315,316]. In rodents, paternal exposure to alcohol similarly increases in the offspring the risk of adverse neurodevelopmental disorders along with FASD-like defects with molecular and physiological alterations [317,318], and it causes various abnormal behavioral phenotypes, including an increase in impulsivity, anxiety- and depressive-like behavior and attention deficit hyperactivity disorder [294,319,320,321] and a decrease in spatiotemporal learning [322] and novelty seeking behavior [323]. There is also evidence that paternal pre-conception alcohol exposure enhances the offspring’s sensitivity to the anxiolytic or motoric effects of alcohol and thus affects its self-administration [316,321,324], and it increases the offspring’s consumption of alcohol [325] and sensitivity to amphetamine-induced hyperlocomotion [326]. Further, paternal alcohol use is shown to impact the brain of the offspring, altering neocortical development, increasing cortical thickness and producing abnormal patterns of gene expression in the neocortex and of intraneocortical connections [319,327]. It additionally increases expression of the dopamine transporter in the frontal cortex [294] and affects an array of hypothalamic genes that mediate neurogenesis and synaptic plasticity during development and direct chromatin remodeling, DNA methylation and posttranslational modifications or transcription regulation [294,316,328,329].

There is growing clinical evidence that paternal pre-conception smoking also produces negative health outcomes inthe offspring, causing birth defects, altering birthweight and increasing the propensity for developing anxiety and depression [330,331]. Similar effects are observed in rodents, with paternal nicotine exposure stimulating locomotor activity of the offspring, impairing their cognitive functions, and increasing their potential for developing attention deficit hyperactivity disorder-like behaviors [332,333,334]. It may also cause a greater aversive response to nicotine in the offspring, leading to a reduction in nicotine reinforcement and self-administration and an attenuation of relapse-related behaviors [317,335]. These behavioral changes are suggested to result from such paternal nicotine-induced disturbances in brain monoamine content and dopamine mRNA expression [332] and also in hippocampal nicotinic acetylcholine receptor binding, evoked cholinergic currents, and the methylation and expression of genes related to neural development and plasticity [335]. Clinical studies demonstrate that paternal use of cannabis similarly has adverse health effects in the offspring, including an increased risk of neural tube defect, heart defects and sudden infant death [310] and an increased transmission of autism-like disorders and schizophrenia [336,337]. In rodent offspring, paternal pre-conception THC exposure produces abnormal locomotor activity, increases impulsivity, impairs cognitive functions and leads to autism-like disorders [317,338], and these behavioral disturbances are suggested to be related to a deficit in dopamine utilization indicative of abnormal synaptic activity [339]. Pre-conception THC exposure in males as well as females alters mRNA expression of cannabinoid, dopamine and glutamatergic receptor genes in the striatum, a key component of the neuronal circuitry mediating compulsive behaviors and reward sensitivity that may increase the risk of neurological and psychiatric disorders in the offspring [340]. Together, these studies underscore how paternal use of these recreational drugs before conception, like maternal pre-conception use, can negatively impact the neurodevelopment and behavioral profile of the offspring.

## 6. Transgenerational Effects Induced by Prenatal Drug Exposure on Behavior and Brain Development

With drug use before conception in females and males as well as maternal drug use during pregnancy having adverse effects on the behavior and brain of the offspring, the next question to consider is whether these effects are long lasting and persist into second or third generations beyond the period of drug use. Both clinical and preclinical studies provide evidence suggesting that such transgenerational effects of drug use, whether by the mother or father, can in fact occur and become evident in future progeny.

### 6.1. Transgenerational Effects of Maternal Drug Exposure on Behavior and Brain Development of Future Generations

Clinical and preclinical studies demonstrate that certain behavioral and neurodevelopmental consequences of maternal alcohol exposure can be transmitted from the first prenatally exposed offspring to their second and third generations of offspring. These transgenerational effects involving a history of alcohol abuse and FASD are described in the grandchildren of grandmothers with documented alcohol use [341]. Similarly, rodent studies involving prenatal alcohol exposure describe multiple behavioral effects lasting into the third generation, including an increase in anxiety- and depressive-like behaviors [342,343], risk-taking behaviors along with abnormal sensorimotor processing [344] and the development and maintenance of an alcohol use disorder [345,346]. These behavioral effects of alcohol are associated with transgenerational changes in brain development, with disturbances in the third generation observed in measures of neocortical development [342], hypothalamic peptide expression and its methylation levels [132,343] and expression of GABA cortical subunits [345]. While the heritability of alcohol dependence is estimated to range from 30–70% [347], epigenetic inheritance as described below is also an important contributing factor, with epigenetic modifications found to be carried across generations and leading to transgenerational phenotype persistence or inheritance [348].

Clinical studies of maternal use during pregnancy of nicotine, with cannabis yet to be studied, similarly reveal major developmental consequences across multiple offspring generations. They demonstrate that grandmaternal smoking during their pregnancy is positively associated with an increased likelihood in their grandchildren of early childhood asthma and diagnoses of autism or attention deficit hyperactivity disorder [349,350]. Further, prenatal nicotine exposure in rodents produces transgenerational effects in the second and third generations, with disturbances observed in behaviors such as hyperactivity and reduced attention span [351] as well as in the brain, pituitary and gonads [352]. Again, these cross-generational effects produced by nicotine may result, in part, from nicotine-induced epigenetic modifications discussed below. Together, these findings illustrate how maternal use of recreational drugs can have long-lasting adverse effects on the brain and behavior, not only of the first prenatally exposed offspring but also of their future generations.

### 6.2. Transgenerational Effects of Paternal Drug Exposure on Behavior and Brain Development of Future Generations

While there is little information in humans on the transgenerational effects of paternal drug use, there are studies in rodents showing that paternal pre-conception exposure to alcohol or nicotine significantly affects the brain development and behavior of the second and third generations of offspring. For example, paternal alcohol consumption causes a variety of deficits, including cognitive function disruptions, hyperactive disorder and alterations in brain growth factors, which persist into subsequent generations [294,318]. Similarly, paternal exposure to nicotine leads to an increase across generations of depressive- and anxiety-like behaviors, locomotor sensitization, cognitive impairments and attention deficit hyperactivity disorder-like behaviors, with these effects diminishing with each successive generation [335,353]. These nicotine-induced behavioral disturbances are accompanied by alterations in monoamine content and dopamine mRNA expression of the offspring brain [332] and also in nicotinic acetylcholine receptor binding, evoked cholinergic currents and methylation and expression of genes related to neural development and plasticity in the hippocampus [335]. This evidence reveals the long-lasting effects that paternal drug use like maternal use can have on future generations of offspring.

### 6.3. Epigenetic Mechanisms Mediating the Transgenerational Effects of Prenatal Drug Exposure

The alcohol-induced epigenetic changes that likely mediate these drug-induced transgenerational effects on behavior and brain development are complex and involve all aspects of cell development. They include alterations in DNA methylation, histone modifications and non-coding RNAs such as microRNA [324,354,355], which in turn affect many genes involved in the cell cycle, apoptosis, migration and formation of neurons and also in the chromatin structure [356,357] and development of brain structures and organs in the embryo [358,359]. The cross-generational effects produced by nicotine may also result from epigenetic modifications, including the deficit in DNA methylation observed in the striatum and frontal cortex of adolescent rodent offspring [360] and alterations in methylation at the promoter region of dopamine receptor gene in the founder’s spermatozoa shown to correlate with changes in its tissue content and expression in the offspring’s brain [332]. Paternal pre-conception cannabis exposure in rodents similarly causes in the father as well as offspring changes in DNA methylation that are related to the possible development of autism-like phenotype [361]. There is further evidence suggesting that the cannabis-induced changes in methylation of genes related to neurodevelopment are permanent, resulting from epigenetic changes in the spermatogonia [361]. These findings with maternal as well as paternal drug use showing transgenerational disturbances in the brain and behavior of the offspring underscore the wide range of effects that can occur and thus raise serious concerns about disturbances that may develop in future generations.

## 7. Sex Differences in Neurological Behaviors and Drug Effects on Brain and Behavior in Humans and Animals

Sex differences in neurological and psychiatric disorders have been difficult to characterize for many reasons. These include the multi-faceted and complex phenomena involved [362,363,364]; the diversity of disorders having wide differences in age of onset, severity and disease trajectory [365]; and the multiple interacting contributions from varied environmental, social and cultural factors, past experiences, and intrapersonal/interpersonal relationships [364,366]. As described below, however, there are specific neurological disorders including SUDs with co-occurring emotional behaviors and associated brain abnormalities that are suggested to be more common in women than men, and there are specific effects of prenatal drug exposure on the brain and behavior related to drug overconsumption showing female offspring to be more vulnerable than males.

### 7.1. Sex Differences in Neurological Disorders and Behaviors Related to SUDs

Whereas some clinical studies reveal no sex differences in neurological disorders [367] and others characterize SUDs as being more prevalent in men [368,369], recent reports suggest this variable evidence results in part from an insufficient power of analysis due to a low inclusion of female subjects [370] and indicate a significant increase in women having SUDs [371,372]. They also describe results with specific measures of drug-related behaviors showing females to be more consistently impacted than males. This is illustrated by sex differences in the “telescoping effect” of drugs, with women showing a faster progression than men from first use to onset of SUDs for alcohol use disorder, daily smoking and cannabis use [373,374,375]. Women are also more vulnerable than men to the neurotoxic effects of drugs such as alcohol, nicotine and cannabis [376,377,378], showing higher incidents of adverse health effects [379,380], and they have less success than men at quitting smoking and alcohol drinking [381,382]. Moreover, the neurological disorders that commonly co-occur with SUDs affect women more disproportionately, and they lead to more severe functional impairments in different aspects of life, with internalizing neurological conditions such as anxiety, depression, eating disorders and schizophrenia more commonly diagnosed in women [14,364,383,384,385]. These disorders complicate SUD treatment outcomes in women more than men, with respect to treatment entry and post-treatment clinical outcomes [14,386,387], and this greater vulnerability is similarly observed during adolescence, as shown by girls with attention deficit hyperactivity disorder having a greater risk than boys of developing SUDs for alcohol, tobacco and cannabis [388]. Studies in rodents confirm this sex difference in behaviors related to drug consumption, showing females to consume higher amounts than males of drugs such as alcohol, nicotine and cannabis [389,390,391] and to be more likely to acquire drug self-administration and reinstate drug seeking behaviors [390,392,393]. Furthermore, consumption of alcohol or nicotine during adolescence leads to greater intake in adult females than males [394,395], and THC has stronger behavioral effects in female than male rodents, producing a greater locomotor suppression and anti-nociception effect [396,397]. These studies provide examples of specific drug-induced behaviors that demonstrate stronger responses in females than males.

### 7.2. Sex Differences in Natural and Drug-Induced Changes in Neural Mechanisms of the Brain

These sex differences in SUDs and related neurological conditions may result from naturally occurring sex differences as well as from sex differences in drug-induced changes in brain structures and neural systems [14,370]. Clinical studies reveal structural differences between drug-naïve girls and boys and drug-naïve women and men in many brain structures involved in reward seeking behaviors and motivation for drugs of abuse [398,399]. They also describe effects of drug use on different brain structures and function that are sex dependent. For example, chronic alcohol consumption in adults causes smaller brain volumes in women more than men [400], and heavy drinking in adolescents with alcohol use disorder reduces the volume of gray matter in the prefrontal cortex consistently more in females than males [401,402]. Nicotine use also has some sex-dependent effects, with female more than male smokers exhibiting a smaller gray matter volume in the amygdala involved in emotion regulation and stress mitigation [403], a finding suggesting that women smoke for different reasons than men [404]. Cigarette-smoking women compared to men also exhibit more neural connectivity within the reward network, possibly related to an increased tolerance that contributes to the greater difficulty experienced by women in quitting smoking [405]. Cannabis use similarly has sex-dependent effects on brain structures, with female users compared to males having a smaller orbitofrontal cortex and cerebellar white matter volumes, areas involved in addiction-related cognitive processes [406,407,408].

These sex differences in the effects of drug use involve multiple biological systems, including neural, hormonal and genetic factors [382,409]. For example, clinical studies of peptide systems such as orexin, known to control complex behaviors related to substance abuse, motivation and reward seeking, reveal sex differences in their role in mediating the development of neurological conditions, with significantly greater changes in their expression reported in female than male patients having an increased risk for depression [410], dementia [411] and Alzheimer’s disease [412]. The neurotransmitter systems, such as dopamine, glutamate and endocannabinoid that mediate the rewarding properties of drugs, are expressed and often exert their effects in a sex-dependent manner [381,385,413], and they are believed to contribute to the sex differences in SUDs involving the overconsumption of alcohol, nicotine and cannabis [90,382]. Examination of the orexin peptide system in rodents demonstrates a greater expression and function in females more than in males and an increased neural activation in response to cocaine cues [414,415]. Moreover, studies of specific molecular mechanisms expressed in the peptide neurons, which as described above involve growth factors, neuroimmune signaling and PPARs that are stimulated by prenatal drug exposure, also indicate sexual dimorphism, with females exhibiting stronger and more robust responses than males [171,416,417]. Together, these clinical and preclinical reports involving adolescent and adult drug exposure provide evidence for sex differences in the development of SUDs and related neurological conditions and in specific neurochemical and molecular mechanisms that likely mediate these behaviors.

### 7.3. Sex Differences in Offspring Produced by Prenatal Drug Exposure on Behavior and Brain Systems Related to SUDs

Whereas some clinical studies have yielded mixed results for sex differences in the effects of prenatal drug exposure due in part to the variability in their timing, dose and methods of self-reporting [418,419,420], there are reports of certain adverse changes consistently showing female offspring to be more vulnerable than males to the impact of drugs such as alcohol, nicotine or cannabis on behavior and brain development. For example, in children prenatally exposed to alcohol, some reports show female progeny more than males to exhibit an increased probability of developing FASD, brain dysmorphology, internalizing or externalizing behaviors, learning impairments and attention deficit hyperactivity disorder, in addition to drinking, smoking and illicit drug use [420,421,422]. Females also present smaller gray matter volumes that are likely to reduce brain plasticity and/or maturation and contribute to their greater behavioral problems [423], and they are found to have a smaller brain volume overall, while males exhibit reduced volume only in specific areas such as the caudate [424]. Further, there are clinical studies describing other sex-dependent, neurodevelopmental impairments after prenatal alcohol exposure, with females more commonly experiencing higher rates of endocrine problems, anxiety and depressive/mood disorders while males present higher rates of attention deficit-hyperactivity disorder, conduct disorder and oppositional defiant disorder [425,426]. While clinical reports of prenatal nicotine exposure yield mixed results for sex differences in the offspring [58,105], there is some evidence that female offspring during adolescence compared to males present thinner brain regions in the cerebral cortex and a greater vulnerability to developing nicotine dependence and an increase in nicotine consumption, again with the male offspring more likely to develop attention deficit hyperactivity disorder [58,418,427,428]. While there are few clinical studies regarding prenatal cannabis exposure, there is evidence that female offspring exposed to cannabis exhibit greater aggression than males and have a higher risk for developing attention deficit hyperactivity disorder [426,429]. In rodent studies of prenatal alcohol exposure, while measures of social behaviors and attention in the offspring describe no sex differences [430,431] or greater social impairments in males [432], reports with other measures demonstrate a greater increase in anxiety and alcohol consumption in female than male offspring [95] and a significant increase only in female offspring of impulsivity and their responsiveness to stress or drug challenges [421,433,434]. While rodent studies of prenatal nicotine exposure provide little evidence for sex-dependent effects [105,141] or show female offspring to be less sensitive to the nicotine-induced increase in anxiety and nicotine consumption [418,435,436,437], studies of prenatal exposure to cannabis describe sex-dependent behavioral effects [197] showing female offspring to be more sensitive than males to the stimulatory effects of cannabis on morphine administration [127,438]. These findings support the idea that certain effects of prenatal drug exposure on behaviors related to neurological disorders such as SUDs are sex-dependent, with female offspring exhibiting a greater vulnerability.

There is limited evidence suggesting a role of specific neurochemical and molecular mechanisms in mediating these sex differences in the behavioral changes induced by prenatal drug exposure. These include the neurochemicals, dopamine, glutamate, GABA and endocannabinoid systems, which control behaviors related to drug consumption and are affected by prenatal drug exposure in a sex-dependent manner [197,435,439,440,441]. They also include neuroimmune systems that control neuronal development [261,262,263] and are suggested in clinical studies to mediate sex differences in neurological disorders such as depressive disorders, autism and attention deficit disorder [442,443]. Consistent with reports showing females to have stronger and more robust adaptive immune responses than males [416], studies in rodents of the chemokine systems detected in hypothalamic peptide neurons [95,268,269], which stimulate the proliferation, migration and projections of these neurons and in turn control alcohol consumption and related behaviors such as anxiety and locomotion [270,271], show them to be stimulated by prenatal alcohol exposure in a sex-dependent manner, with female offspring more strongly affected than males [95,171,273]. In addition, the behavioral changes that accompany these neural effects, including an increase in alcohol consumption and anxiety, are also significantly greater in females than males [95]. Consistent with studies in the periphery [444,445], the fibroblast growth factor-2 expressed in the hypothalamic peptide neurons similarly exhibits sex differences, with prenatal alcohol exposure in rodents increasing only in female offspring its expression in these neurons and their total number of nuclear transcripts, effects suggested to contribute to the greater increase in neurogenesis and alcohol consumption observed in females [259]. Notably, these sex differences in the effects of alcohol are similarly observed in zebrafish, with embryonic alcohol exposure causing a greater stimulation of different peptide neurons in the female than male offspring [103]. Whereas sex differences in the effects of prenatal nicotine and cannabis exposure on these brain systems have yet to be characterized, this strong evidence involving prenatal alcohol exposure suggests that these drugs may have similar sex-dependent effects on the offspring that contribute to the sex differences in their behavioral changes related to the overconsumption of drugs.

## 8. Conclusions

The use of recreational drugs by teenagers and during pregnancy occurring widely throughout the world has increased markedly at younger ages in recent years. For example, studies show that drug use by 8th graders has increased by 61% from 2016 to 2020 [446], and the number of secondary students vaping nicotine in the prior month has nearly doubled since 2017 [447]. This rise in drug use has resulted, in part, from the increase in availability of nicotine that can be used through vaping in almost any setting, the legalization of cannabis that can also be vaped and the marked increase in number of smoke shops where vaping materials are sold [448,449]. Along with the increased availability and use by teenagers, however, there is a lack of knowledge about the serious and wide range of adverse effects that drug use can have. While most students in the US learn about drugs with an abstinence-only logic base as reported by the Drug Policy Alliance, this advice is often unsuccessful and fails to address other important information that students need to learn [450]. For example, while teens hear about the harmful effects drug use can have on their own body, they are told less about the effects that drugs can have on their brain and how these, in turn, can lead to an increased risk of developing neurological disorders such as SUDs and their co-occurring conditions. Also, while women are warned about the adverse effects that alcohol and nicotine use can have during pregnancy, they are less aware of the effects that cannabis use can have, and they also receive little information about the serious effects that drug use has in men. Further confusing for teenagers are the marked increase in number of smoke shops and communities legalizing cannabis and the marketing of vaping as a healthy alternative to smoking cigarettes, leading them to believe there is little danger or harm in occasionally vaping nicotine or cannabis.

The clinical and preclinical studies described in this report provide strong evidence that drug use during adolescence and pregnancy can produce long-lasting changes in neurological disorders and can also affect brain mechanisms that are functionally and causally related to the drug-induced disturbances in behavior such as an increased risk of developing SUDs. In addition to revealing behavioral changes in rodents and even zebrafish that are similar to those described in clinical reports, recent studies of drug effects on the brain in animals are characterizing in considerable detail a wide range of disturbances in neuronal systems, their neurocircuitry and intracellular molecular signaling, which may underlie these behavioral changes. The evidence summarized in this report, often showing the three drugs, alcohol, nicotine and cannabis, to have similar adverse effects on different measures of neuronal development and behavior, provide support for the following conclusions regarding the range of disturbances that result from drug use by young and more vulnerable individuals. These conclusions are: (1) Drug use during adolescence has long-lasting, harmful effects on their future lives, producing changes in their brain and increasing their risk of developing neurological conditions. (2) Maternal use of these drugs during pregnancy has diverse negative effects on the brain and behavior of the offspring. (3) These harmful effects of drug use during adolescence and pregnancy can occur even at low doses of the drugs. (4) They are produced in the offspring by paternal as well as maternal drug use before conception. (5) These effects of drug use at these vulnerable ages can even be passed onto future generations. (6) The effects of drug use may exhibit sex differences, with females more vulnerable than males to developing SUDs with co-occurring emotional behaviors and showing disturbances in the brain that may mediate these behavioral changes.

With research in this report providing solid support for these conclusions, the information they provide needs to be used to educate the young population, both women and men, about the harmful effects of drug use. The results described should allow them to carefully weigh the risks and benefits of drug use and make informed decisions themselves as to when and how often to use the drugs. It should additionally help them to set realistic goals for managing their drug use rather than just quitting drug intake altogether, while also encouraging research on new therapeutic strategies that might help them to limit their drug use.
